# The in Vitro and in Vivo Antitumor Activities of Tetracyclic Triterpenoids Compounds Actein and 26-Deoxyactein Isolated from Rhizome of *Cimicifuga foetida* L.

**DOI:** 10.3390/molecules21081001

**Published:** 2016-07-30

**Authors:** Desong Wu, Qi Yao, Yajuan Chen, Xiaodong Hu, Chen Qing, Minghua Qiu

**Affiliations:** 1 School of Pharmaceutical Science & Yunnan Key Laboratory of Pharmacology for Natural Products, Kunming Medical University, Kunming 650500, China; wudesong14@163.com (D.W.); chenyajuan0873@163.com (Y.C.); 2 Department of Pharmacology, Yunnan Institute of Materia Medica, Kunming 650111, China; yaoqi313@126.com; 3 Department of Histology and Embryology, Bengbu Medical College, Bengbu 233030, China; huxiaodongzhi@126.com; 4 Kunming Institute of Botany, Chinese Academy of Sciences, Kunming 650201, China; qiuminghua@mail.kib.ac.cn

**Keywords:** actein, 26-deoxyactein, antitumor activity, IC_50_, cell cycle

## Abstract

Aims: This work aims to study the in vitro and in vivo antitumor activities of tetracyclic triterpenoids compounds actein and 26-deoxyactein. Further, the mechanism is investigated. Methods: In vitro, a modified MTT method was used to assay the cytotoxicities of actein and 26-deoxyactein in 12 human tumor cell lines. In vivo, mouse sarcoma S180 and human lung cancer A549 cells were respectively implanted subcutaneously in ICR mice and nude mice to establish implanted tumor models. Flow cytometry (FCM) was used to assay the cycle distribution of the tumor cells. Immunohistochemistry was used to measure CD31-positive expression in the xenogrft tumor by analyzing microvessel density (MVD). In addition, acute toxicities of actein and 26-deoxyactein were also evaluated. Results: Actein and 26-deoxyactein inhibited the proliferation of the 12 human cancer cell lines tested with the values of 50% inhibitory concentrations (IC_50_) between 12.29 and 88.39 μg/mL. In vivo, both actein (3–27 mg/kg) and 26-deoxyactein (3–27 mg/kg) significantly inhibited the growth of the implanted sarcoma S180 in a dose-dependent manner. Actein (10, 30 mg/kg) and 26-deoxyactein (10, 30 mg/kg) markedly inhibited the xenograft growth with T/C (%) values of 38%, 55% for actein, and 35%, 49% for 26-deoxyactein. Compared with the vehicle control, actein (10, 30 mg/kg) and 26-deoxyactein (10, 30 mg/kg) significantly reduced the MVD in the xenograft tumor. The FCM result showed that human leukemia HL-60 cells were arrested at G_1_ phase after treated with either actein (6.25–25 μg/mL) or 26-deoxyactein (6.25–25 μg/mL) for 48 h. A limited trial in mice showed that both of the minimal lethal doses (MLDs) of actein and 26-deoxyactein were over 5 g/kg. Conclusions: Both actein and 26-deoxyactein have low toxicities. Importantly, both these two tetracyclic triterpenoids compounds isolated from rhizome of *Cimicifuga foetida* L. have significant antitumor activities in vitro and in vivo, which is associated with cell cycle arrest and angiogenesis inhibition.

## 1. Introduction

Cancer is a disease seriously threatening human life and health [1]. According to the statistical data in 2011, there were over 600 million people who die of malignancies yearly worldwide, with other 130 million in China [2]. Although chemotherapy plays a key role in the treatment of cancer, in the clinic, there still remains some disadvantages such as low selectivity, toxicity, adverse effects, and so on. Thus, it is urgent to search for optical chemotherapeutic agents with high activities and low toxicities.

**Figure 1:**
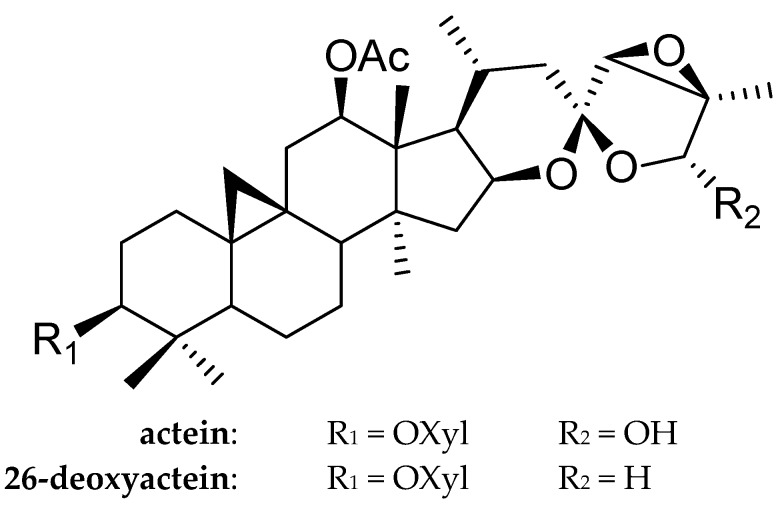
Chemical constructures of actein and 26-deoxyactein.

Actein and 26-deoxyactein are tetracyclic triterpenoids compounds isolated from the rhizome of *Cimicifuga foetida* L. (Figure 1). In North America, the *cimicifuga* species had a long medicinal history, mainly used to treat diarrhea, sore throat, and rheumatism [3]. Currently, about 200 compounds such as saponins including *cimigenol*, actein, 26-deoxyactein and cinnamic acid derivatives like ferulic acid, iso-ferulic acid, caffeic acid have been isolated from the *cimicifuga* herbs [4,5,6]. Among these compounds, the saponins were documented to have antiviral, antitumor, anti-inflammatory and analgesic, immune regulatory activities [7].

Recently, people have focused on pharmacological activities of the *cimicifuga* herbs. Extracts derived from the rhizome of the *cimicifuga* herbs have been applied in clinic in Germany to treat female-related diseases such as menopause, and estrogen disorders caused by surgical removal of ovaries or uterus. Thus, the *cimicifuga* herbs are a class of natural medicinal plants with potential medicinal values. Tian et al. found that 24-*O*-acrtylcimigenol -3-*O*-β-d-xylopyranoside significantly inhibited the HepG-2 cell proliferation and arrested cell cycle at G_2_/M phase [8]. Additionally, total glycosides from the *cimicifuga* herbs markedly inhibited the growth of HepG-2 in vitro and implanted mouse H22 hepatoma in vivo [9].

Based on the previous studies of others [8,9], in the present study the antitumor activities of actein and 26-deoxyactein were assessed in different cancer cell lines in vitro and the S180 cell-implanted model and the A549 xenograft model in vivo. Furthermore, the mechanisms including cell cycle distribution and angiogenesis were also studied. In addition, the preliminary safety evaluation for these two compounds was performed. In view of this, we hope to clarify the clinical potential of actein and 26-deoxyactein in the treatment of malignancies.

## 2. Results

### 2.1. Both Actein and 26-Deoxyactein Inhibited the Growth of the 12 Human Tumor Cell Lines Tested

**Figure 2:**
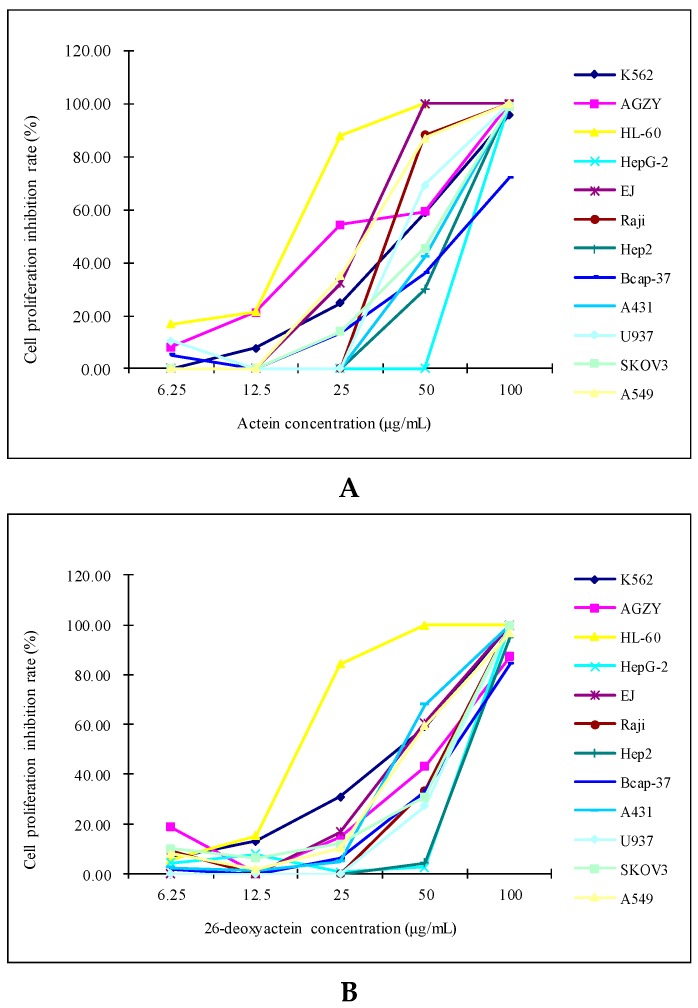
The growth inhibitory effects of actein and 26-deoxyactein on the 12 human tumor cell lines tested. (**A**) The growth inhibitory effect of actein on the 12 human tumor cell lines tested; (**B**) The growth inhibitory effect of 26-deoxyactein on the 12 human tumor cell lines tested.

The results showed that both actein and 26-deoxyactein inhibited the growth of the 12 human tumor cell lines tested in concentration-dependent manners. With the increases in the concentrations of actein and 26-deoxyactein, cell proliferation inhibition rates were higher and higher (Figure 2).

The IC_50_ values of actein and 26-deoxyactein for the HL-60 were 12.29 and 14.54 μg/mL, which were respectively lowest in the 12 tested cell lines, suggesting a high susceptibility of this cell line to these two compounds (Table 1). In addition, actein and 26-deoxyactein inhibited other cell lines with the IC_50_ values between 19.35 and 22.15 μg/mL (Table 1).

### 2.2. Actein and 26-Deoxyactein Arrest the HL-60 Cells at G1 Phase

**Figure 3:**
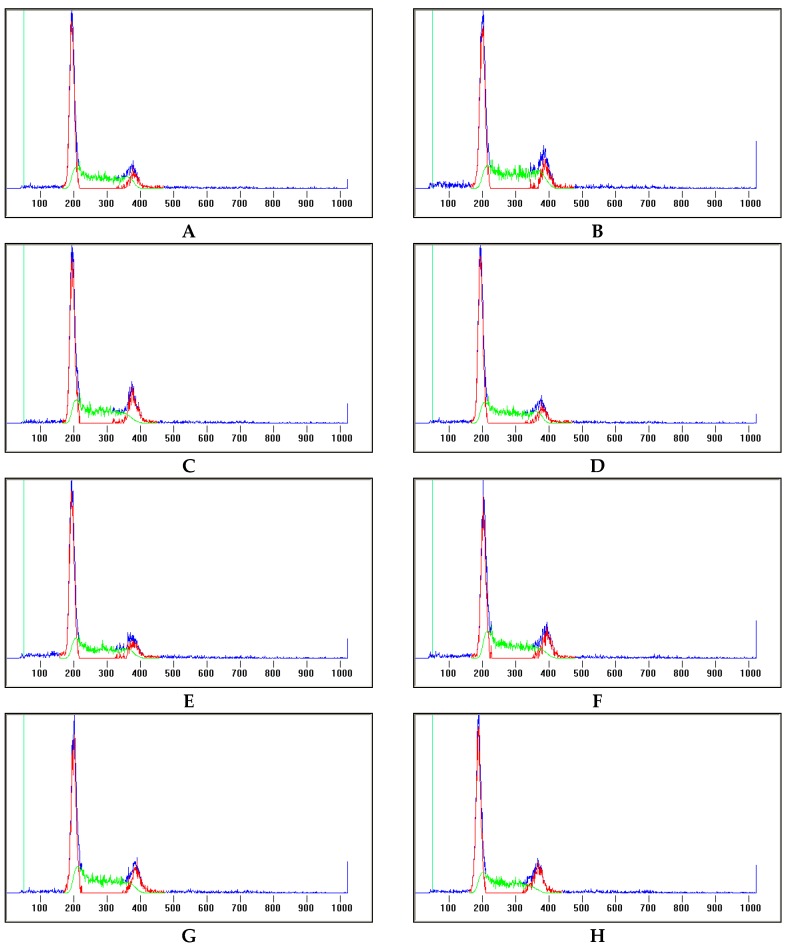

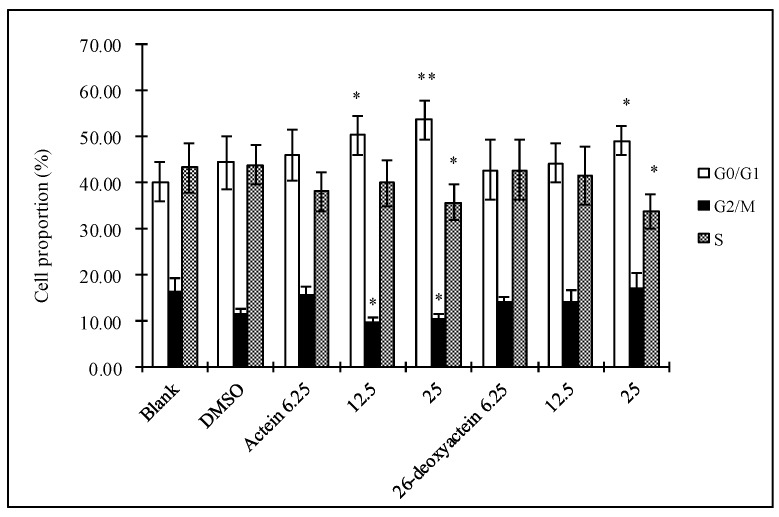
Cell cycle distribution of the HL-60 cells ($\bar{x}$ ± s, *n* = 3). (**A**) Cell cycle distribution of the HL-60 cells without treatment; (**B**) Cell cycle distribution of the HL-60 cells treated with vehicle (DMSO); (**C**) Cell cycle distribution of the HL-60 cells treated with actein at a final concentration of 6.25 μg/mL; (**D**) Cell cycle distribution of the HL-60 cells treated with actein at a final concentration of 12.5 μg/mL; (**E**) Cell cycle distribution of the HL-60 cells treated with actein at a final concentration of 25 μg/mL; (**F**) Cell cycle distribution of the HL-60 cells treated with 26-deoxyactein at a final concentration of 6.25 μg/mL; (**G**) Cell cycle distribution of the HL-60 cells treated with 26-deoxyactein at a final concentration of 12.5 μg/mL; (**H**) Cell cycle distribution of the HL-60 cells treated with 26-deoxyactein at a final concentration of 25 μg/mL. * *p* < 0.05, ** *p* < 0.01 vs. DMSO.

The cell proportions in the G_1_ phase were respectively 46.17%, 50.45%, 53.67% after treatment with actein for 48 h at 6.25, 12.5, 25 μg/mL. Correspondingly, the cell proportions in the G_1_ phase were respectively 42.91%, 44.37%, and 49.14% after treatment with 26-deoxyactein (6.25–25 μg/mL) for 48 h. The cell number in the G_1_ phase increased gradually along with the increases in the concentrations of these two compounds (Figure 3). Accordingly, the cell proportions in the G_2_/M and S phases were significantly reduced after either the actein or 26-deoxyactein treatment (Figure 3).

### 2.3. Both Actein and 26-Deoxyactein Inhibit the Growth of Implanted S180 in the Mice

**Figure 4:**
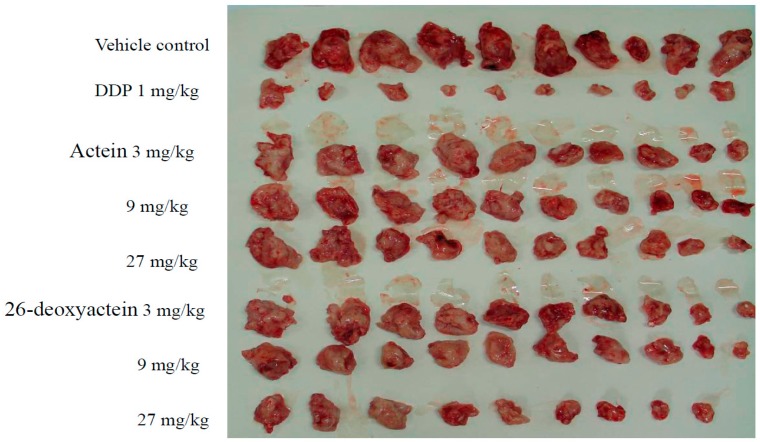
Implanted S180 tumor tissues in groups.

The implanted S180 tumor growth in the DDP-treated mice was significantly inhibited with an inhibitory rate of 88.87%. The tumors in the actein- and 26-deoxyactein group were respectively smaller than that in the vehicle group (Figure 4). The growth inhibition rate of the implanted S180 were respectively 40.24%, 44.27%, and 52.80% after treatment with actein (3, 9, 27 mg/kg). Correspondingly, the inhibition rates in the 26-deoxyactein (3, 9, and 27 mg/kg) group were respectively 40.80%, 52.59%, and 68.66% (Table 2). From the results, we concluded that, in vivo, both actein and 26-deoxyactein had significant antitumor activity, and 26-deoxyactein was slightly stronger than actein.

### 2.4. Actein and 26-Deoxyactein Inhibit the Growth of the Implanted A549 Tumor in the Nude Mice

**Figure 5:**
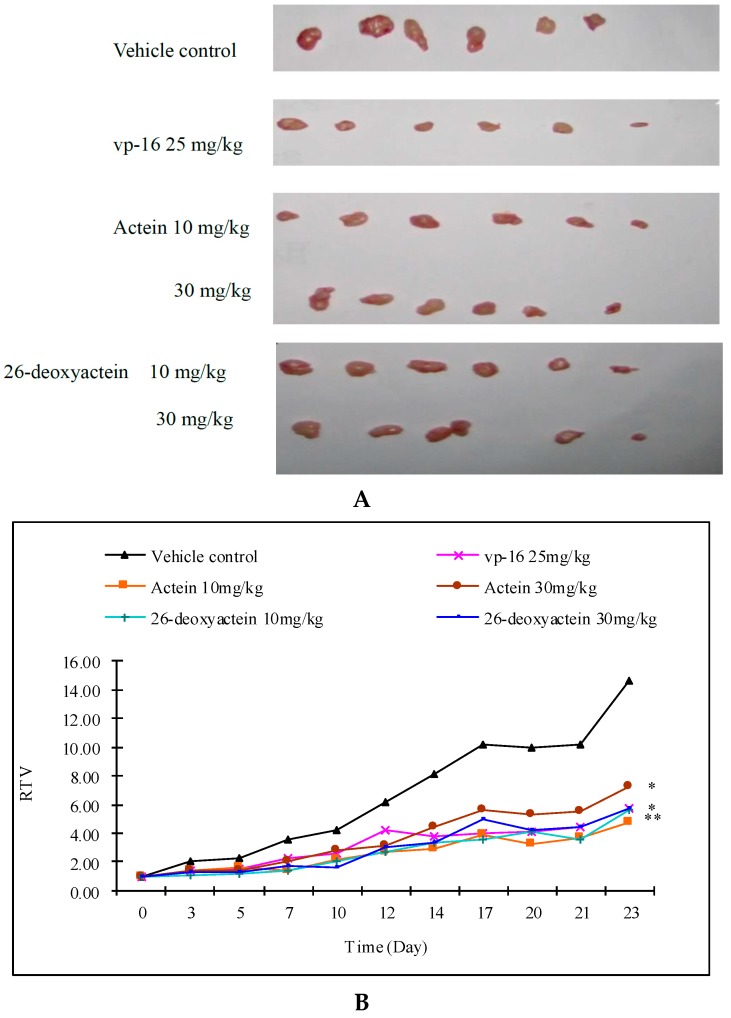

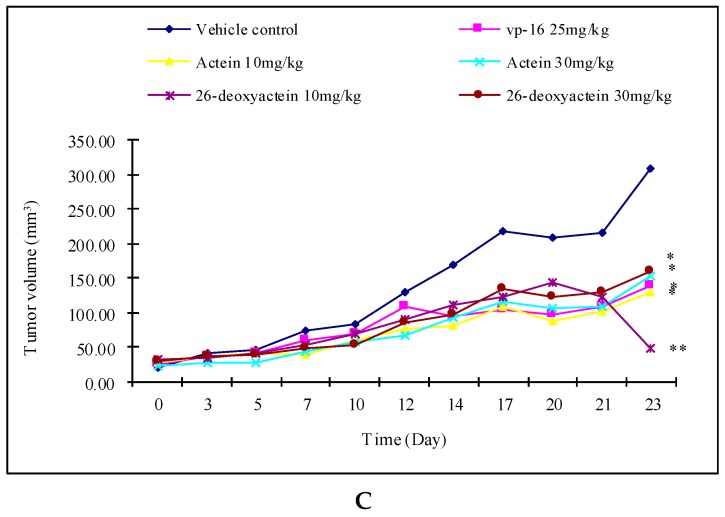
The growth of implanted human lung cancer A549 in the nude mice ($\bar{x}$ ± *s*, *n* = 6). (**A**) Implanted tumor after peeled off; (**B**) RTV at specific time points; (**C**) Tumor volume at specific times.

Compared with the vehicle control, VP-16 (25 mg/kg) significantly inhibited the growth of the implanted A549 cells in the nude mice with a T/C (%) value of 39.09%, and the difference was significant. Actein (10, 30 mg/kg) and 26-deoxyactein (10, 30 mg/kg) markedly inhibited the growth of the implanted A549 with T/C (%) values of 38%, 55% for actein, and 35%, 49% for 26-deoxyactein (Figure 5).

### 2.5. Both Actein and 26-Deoxyactein Downregulate Cd31-Positive Expression in the Implanted Tumor of the Nude Mice

**Figure 6:**
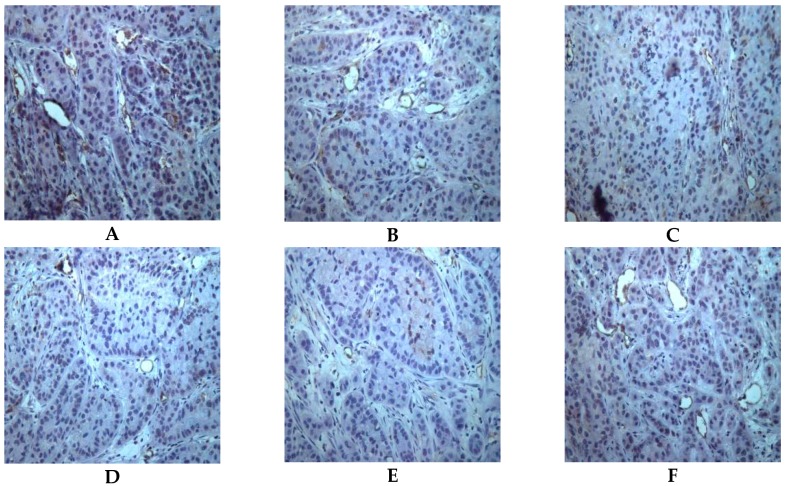

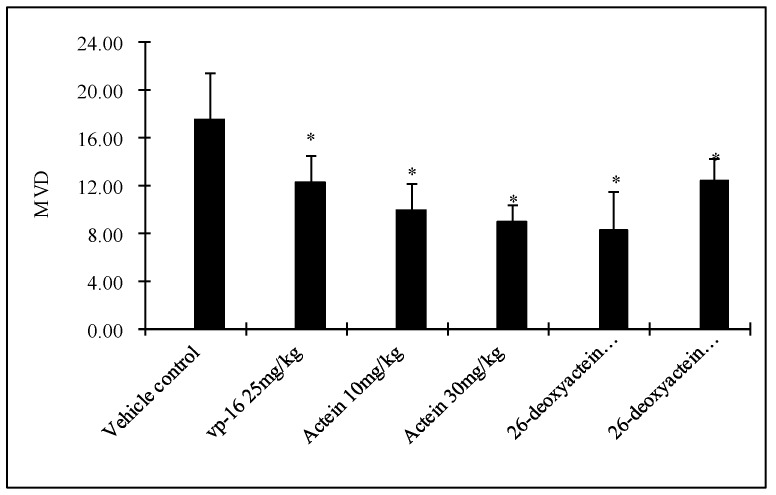
CD31-positive expression in the xenograft tumor tissues in the nude mice ($\bar{x}$ ± *s*, *n* = 6) (SABC × 200). (**A**) CD31-positive expression in the vehicle control group; (**B**) CD31-positive expression in the VP-16-treated group; (**C**) CD31-positive expression in the low-dose actein group (10 mg/kg); (**D**) CD31-positive expression in the high-dose actein group (30 mg/kg); (**E**) CD31-positive expression in the low-dose 26-deoxyactein group (10 mg/kg); (**F**) CD31-positive expression in the high-dose 26-deoxyactein group (30 mg/kg).

Actein or 26-deoxyactein (10–30 mg/kg) significantly reduced the CD31-positive expression in the implanted tumor tissue. Alternatively, actein or 26-deoxyactein reduced the MVD compared with the vehicle control (Figure 6). The microvessel distribution in the actein or 26-deoxyactein group was sparser than that in the vehicle group (Figure 6).

### 2.6. Preliminary Safety Evaluation of Actein and 26-Deoxyactein

Four hours after the administrations, reduced activity, eating, and drinking was observed in the majority of the animals. Also, weight loss was significant 3 and 7 days after the treatment of actein and 26-deoxyactein (Table 3). Actually, there was no significant difference in body weight among the treated groups and the vehicle group on the 14th day (Table 3). Within the 14-day observation period, all the animals survived the administrations of actein and 26-deoxyactein at a total dose of 5 g/kg. These findings showed the minimal lethal dose of actein or 26-deoxyactein is over 5 g/kg.

Further, the autopsy result showed that the colors of the liver and kidney became shallow and the lung was congestive in some of the treated animals (Table 4). Based on this, we speculated that lung, liver, and kidney were involved in the acute toxicity caused by actein or 26-deoxyactein.

## 3. Discussion

Actein and 26-deoxyactein are tetracyclic triterpenoids compounds isolated from rhizome of the *cimicifuga* herbs with a wide range of biological activities including antitumor activity [8,9], but no further systematic and comprehensive study of their antitumor activities has been documented.

In our previous preliminary study, we found that actein and 26-deoxyactein significantly inhibited the growth of the implanted S180 sarcoma in the mice. To confirm antitumor activities and the possible mechanisms of these two compounds, we selected actein and 26-deoxyactein in the therapy of an allogeneic mouse model and a human xenograft tumor model. Further, cell cycle and angiogenesis were also studied.

Tumourigenesis is a result of cell cycle disorganisation, leading to uncontrolled cell proliferation and cancer progression [10,11]. Normally, cell cycle-related genes and their products such as cyclins [11,12,13], cyclin dependent kinases (Cdks) [12,13,14,15], Cdk inhibitors (CKI) [14,15] and extra cellular factors (i.e., growth factors) [11] were involved in the cell cycle regulation. Also, the modulation of the cell cycle is of importance in current applications and prospects for future development of chemotherapeutic reagents [14,16]. Candidate targets regulating cell cycle involve G_1_ to S phase or G_2_ to M phase transition. In the present study, the cell cycle distribution of the HL-60 was assayed after the actein or 26-deoxyactein treatment. It revealed that the cell proportion in the G_1_ phase increased and the cell proportions in G_2_/M and S phases were reduced, suggesting G_1_ arrest induced by these two tetracyclic triterpenoids compounds.

To the best of our knowledge, biological characteristics of animal-derived tumors are distinct to that of human beings. Generally, animal-derived tumors are more malignant with more rapid growth rates. Thus, in order to more objectively evaluate the antitumor activities of actein and 26-deoxyactein, both allogeneic and xenograft implanted tumor models were used in the present study. The results suggested that both actein and 26-deoxyactein inhibited the implanted S180 and A549 tumor cells in vivo, which were in line with the in vitro results to some degree.

Furthermore, the role of microvessel angiogenesis was assayed in the therapeutic effects of actein and 26-deoxyactein in the xenograft implanted tumor model. Generally, tumor metastasis will not occur in the absence of angiogenesis [17,18]. Angiogenesis provides growth nutrients and excretes metabolism products, promoting vascular leakage and tumor metastasis. Angiogenesis therefore is a basis for sustained growth, invasion and metastasis of tumor [17,18].

Microvessel density (MVD) is one of the key indices to assess the therapeutic efficacy of angiogenesis inhibition. Commonly, endothelial cell specific biomarkers can be used to identify the neovascularization by immunohistochemistry techniques. CD31, also named platelet endothelial cell adhesion molecule (PECAM-I), is an endothelial adhesion molecule, mediating a variety of cell biological processes [19,20,21]. It has been confirmed that CD31 is a marker that can transform tumor cells to endothelial cells. Our findings revealed that actein and 26-deoxyactein significantly reduced microvessel density (MVD) and microvessel distribution, which might be responsible for the therapeutic efficiency of actein and 26-deoxyactein in vivo.

The weight loss was significant 3 and 7 days after the treatment of actein or 26-deoxyactein. Actually, there was no significant difference in the body weight among the treated groups and the vehicle group on the 14th day. We speculated that at the beginning of administration, the blood drug concentration in the body was comparatively high. Thus, the toxicity of the drug was present at the early stage, which inhibited the growth of the animals. In time, the drug was gradually eliminated by the liver, kidney, and so on. Therefore, there was no significant effect on body weight when the blood drug concentration of actein or 26-deoxyactein in the body was low.

Taken together, actein and 26-deoxyactein had significant antitumor activities in vitro and in vivo. Also, the mechanism is associated with cell cycle arrest and tumor angiogenesis inhibition.

## 4. Materials and Methods

### 4.1. Cell Lines and Main Reagents

Cells HL-60 (human promyelocytic leukemia cell line), U937 (human histiocytic lymphoma cell line), Raji (human Burkitt's lymphoma cell line), K562 (human chronic myelogenous leukemia cell line), A549 and AGZY (human lung adenocarcinoma cell lines), Hep2 (human laryngocarcinoma cell line), Bcap37 (human breast cancer cell line), A431 (human Basal cell carcinoma cell line), EJ (human bladder cancer cell line), HepG-2 (human hepatic carcinoma cell line), SKOV3 (human ovarian cancer cell line), and S180 (mouse sarcoma cell line) were provided by Shanghai Institutes for Biological Sciences, Chinese Academy of Sciences (CAS) (Shanghai, China). All the cells were cultured in roswell park memorial institute (RPMI) 1640 medium supplemented with 10% fetal bovine serum (FBS) and grew at 37 °C in an atmosphere of 5% CO_2_. When the cells reached a confluence of approximately 90%, they were then digested and passaged every 3 days. The cells in passages 3–5 were used for experimental analyses.

Actein and 26-deoxyactein were provided by Prof. Minghua Qiu, Kunming Institute of Botany, CAS (Kunming, China). CD31 goat anti-mouse IgG monoclonal antibody was purchased from Santa Cruz Biotechnology Co., Ltd. (Shanghai, China).

### 4.2. Experimental Animals

SPF female ICR mice weighting 18–22 g were provided by Experimental Center, Kunming Medical University (Kunming, China). SPF male BALB/c nude mice aging 5–6 week were purchased from Shanghai Institute of Materia Medica, CAS (Shanghai, China). The animals were given free access to food and water. All the experiments were conducted in accordance with the national guidelines for the care and use of laboratory animals. This study was approved by the Ethnic Committee of Kunming Medical University.

### 4.3. Cell Proliferation Assay

The cell proliferation assay was performed by the modified MTT method. Briefly, the cells in passages 3–5 (5 × 10^4^–1 × 10^5^/mL) in exponential phase were seeded in 96-well plates. After cultured for 0 h (for suspended cells) or 24 h (for adherent cells), actein and 26-deoxyactein were respectively added to respective wells to reach various concentrations. The cells were then cultured for another 48 h (for suspended cells) or 72 h (for adherent cells). After that, 20 μL of MTT (5 mg/mL) was then added in each well followed by the adding of 100 μL of triple liquid containing 1.2 mL 36%–37% concentrated hydrochloric acid, 100 g SDS, and 50 mL isobutanol dissolved in 1 L triple-distilled water. After culturing for 12 h, the cell viability was assessed by measuring the optical density (OD) at 570 nm in a Multiskan™ GO Microplate Spectrophotometer (Thermo Fisher Scientific, Waltham, MA, USA). Data from triplicate samples were averaged and plotted on a growth curve. The cell proliferation inhibition rate was calculated in accordance with the following formula: Cell proliferation inhibition rate (%) = (OD_control_ − OD_treated_)/OD_control_ × 100%.

### 4.4. Cell Cycle Assay

The treated or untreated cells (1 × 10^5^) were cultured in the RMPI 1640 supplemented with 10% FBS for 3 days. After that, the cells were digested by 0.25% trypsin and collected. Then the cells were centrifuged at 1000 rpm for 5 min at 4 °C. The pellet was resuspended in 0.01 M PBS. After that, the cells were fixed in 70% cold ethanol at 4 °C for 24 h followed by a co-incubation with RNAase and then labeled with propidium iodide (PI). The cell cycle was analyzed by using a flow cytometry (FCM) (Olympus, Tokyo, Japan) at 488 nm excitation wavelength and 530 nm emission wavelength.

### 4.5. Establishment of the S180 Cell-Implanted Tumor Mouse Model and Treatments

Eighty female ICR mice were subcutaneously inoculated with the S180 cells (1 × 10^7^). Twenty-four hours after the inoculation, the animals were randomly divided into a vehicle group, a diamminedichloroplatinum (DDP) group, three actein-treated groups (3, 9, 27 mg/kg) and three 26-deoxyactein-treated groups (3, 9, 27 mg/kg). Ten animals were in each group. Actein and 26-deoxyactein were dissolved in 0.5% sodium carboxyl methyl cellulose (CMC-Na). The animals in the actein- or 26-deoxyactein-treated groups were administrated with various doses of actein or 26-deoxyactein. Correspondingly, the animals in the vehicle group were administrated with equal volumes of 0.5% CMC-Na. The mice in the DDP group were injected with DDP (1 mg/kg) intraperitoneally in accordance with the body weight.

The mice in the groups received various treatments for successively 10 days and once a day. At the end of the experiment, the animals were sacrificed by cervical dislocation. Tumor tissue was then peeled off and weighed up. The tumor inhibition rate was also calculated.

### 4.6. Establishment of the A549 Xenogrft Tumor Model and Treatments

Male BALB-c nude mice were subcutaneously inoculated with 0.2 mL of the prepared cell suspension (2 × 10^7^/mL). Subsequently, the tumor formation was successively observed and recorded. When the tumor volume reached 100~300 mm^3^, the animals were randomly divided into a vehicle group, an etoposide (VP-16) group (25 mg/kg), two actein groups (10, 30 mg/kg) and two 26-deoxyactein groups (10, 30 mg/kg) (6 animals in each group). The mice in the groups received various treatments for successively 6 days and withdrawal for one day, and this whole experiment lasted for 23 days.

The general conditions of the animals, including mention, diet, activity, and etc. were observed and recorded. Tumor volume (TV) was observed and recorded at specific time points to plot a growth curve [10,11]. Meanwhile, relative tumor volume (RTV) was also counted.

TV = 1/2 × a × b^2^ (1)

where a, b represent long and short diameter of the tumor tissue, respectively;

RTV = V_t_/V_0_ (2)

where V_0_ represents original tumor volume at the beginning of the administration (day 0), and V_t_ represents the tumor volume at different measurement time points;

T/C (%) = T_RTV_/C_RTV_ × 100% (3)

where T_RTV_ represents RTV of the treated group, C_RTV_ represents RTV of the control group.

Criteria are judged by the criteria of Fodstad: >50%, inactivity (−); ≤50%, marginal activity (+/−); ≤40%, moderate activity (+); ≤25%, high activity (++); ≤10%, particularly strong activity (+++).

### 4.7. Immunohistochemistry Assay of CD31-Positive Expression in the Xenograft Tumor Tissue in the Nude Mice

After weighing, the xenograft tumor tissues were collected and cut into a size of approximately 3.0 × 1.0 mm and fixed in paraform for 12 h followed by dehydration and embedding in paraffin. Then, the paraffins were consecutively cut into 4 μm-thick sections.

To measure the microvessel density (MVD) in the tumor tissues, the sections were co-cultured with goat anti-mouse monoclonal CD31 antibody. For quantification of positively stained vessels, the number of microvessel was counted in 10 randomly chosen high-power fields by two independent reviewers.

### 4.8. Preliminary Safety Valuation of Actein and 26-Deoxyactein

Limit trail was used to assess preliminary safety of actein and 26-deoxyactein. Briefly, 40 ICR mice with an equal sex ratio (1:1) were randomly divided into an actein group and a 26-deoxyactein group (20 animals in each group). The animals were administrated with actein or 26-deoxyactein at a dose of 5 g/kg once, respectively. After the administrations, the general status of the animals was observed for 14 days and the body weight was recorded at specific time points. In addition, a vehicle group (20 animals including 10 female and 10 male) was also selected in the present study.

### 4.9. Data Presentation and Statistical Analysis

All the data are expressed as mean ± S.E.M. The data were performed by SPSS13.0 statistics software (SPSS Inc., Chicago, IL, USA). The significance of differences between groups was evaluated by one-way analysis of variance (ANOVA) for multiple comparisons. A *p* value of less than 0.05 was considered to be significant.

## Figures and Tables

**Table 1:** IC_50_ (ug/mL) of actein and 26-deoxyactein for the 12 human tumor cell lines.

Cell Line	Actein (μg/mL)	26-Deoxyactein (μg/mL)
K562	39.83	22.15
AGZY	19.35	54.49
HL-60	12.29	14.54
HepG-2	50–100	42.43
EJ	29.14	38.04
Raji	45.4	52.72
Hep2	52.22	88.39
Bcap-37	79.17	71.04
A431	60.28	29.86
U937	47.08	52.61
SKOV3	44.71	26.36
A549	34.68	37.72

**Table 2:** Tumor weight and growth inhibition in the tumor-bearing mouse model implanted with S180 cells ($\bar{x}$ ± *s*, *n* = 10).

Group	Dosage (mg/kg)	Tumor Weight (g)	Growth Inhibition (%)
Vehicle control	−	2.83 ± 0.91	−
DDP	1	0.31 ± 0.22 **	88.87
Actein	3	1.69 ± 0.83 *	40.24
	9	1.58 ± 0.79 *	44.27
	27	1.34 ± 0.74 *	52.80
26-Deoxyactein	3	1.68 ± 0.84 *	40.80
	9	1.34 ± 0.70 *	52.59
	27	0.89 ± 0.49 *	68.66

* p < 0.05, ** *p* < 0.01 vs. Vehicle.

**Table 3:** Body weight (g) of mice in groups within 14 days of administrations ($\bar{x}$ ± s, *n* = 20).

Group	Dosage (g/kg)	Body Weight (g)			
		Time after Administration (Day)			
		0	3	7	14
Vehicle	−	18.9 ± 0.8	22.1 ± 1.3	25.9 ± 1.5	28.9 ± 1.9
Actein	5.0	18.4 ± 0.7	19.8 ± 1.4 **	24.6 ± 1.7 *	27.7 ± 2.9
26-Deoxyactein	5.0	18.8 ± 0.8	19.6 ± 1.2 **	24.4 ± 2.0 *	27.6 ± 3.2

* *p* < 0.05, ** *p* < 0.01 vs. Vehicle.

**Table 4:** Incidence of toxicity in liver and kidney after oral administration of actein or 26-deoxyactein.

Group	Dosage (g/kg)	Liver Toxicity		Kidney Toxicity		Lung Toxicity	
		Present	Absent	Present	Absent	Present	Absent
Vehicle	−	0	20	0	20	0	20
Actein	5.0	2	18	1	19	2	18
26-Deoxyactein	5.0	2	18	2	18	1	19
